# Perception of pregnant individuals, health providers and decision makers on interventions to cease substance consumption during pregnancy: a qualitative study

**DOI:** 10.1186/s12889-024-18397-x

**Published:** 2024-04-09

**Authors:** Andrea Vila-Farinas, Mónica Pérez-Ríos, Agustín Montes-Martínez, Jasjit S- Ahluwalia, Nerea Mourino, Julia Rey-Brandariz, Yolanda Triñanes-Pego, Cristina Candal-Pedreira, Alberto Ruano-Ravina, Patricia Gómez-Salgado, Carmen Miguez-Varela, María Tajes-Alonso, Isabel Loureiro-Fuentes, Juan Riesgo-Martín, Araceli Valverde-Trillo, Isabel Fernández-Lema, Mercedes Rey-Arijón, Isabel Freiría-Somoza, María Rodríguez-Pampín, Leonor Varela-Lema

**Affiliations:** 1 Department of Preventive Medicine and Public Health, University of Santiago de Compostela, A Coruna, Spain; 2 Consortium for Biomedical Research in Epidemiology and Public Health, [CIBER en Epidemiología y Salud Pública/CIBERESP], A Coruna, Spain; 3 Health Research Institute of Santiago de Compostela [Instituto de Investigación Sanitaria de Santiago de Compostela - IDIS], A Coruna, Spain; 4 Departament of Behavioral and Social Sciences, Brown University School of Public Health, Providence, RI USA; 5 Scientific-technical Assessment Unit [Avalia-t]. Galician Health Knowledge Management Agency, Agencia Gallega de Conocimiento en Salud/ACIS, A Coruna, Spain; 6 Department of Clinical Psychology and Psychobiology, University of Santiago de Compostela, A Coruna, Spain; 7 Mental Health Department, Regional Health Authority, Galician Regional Authority [Xunta de Galicia], A Coruna, Spain; 8 Ordes Health Center, Galician Health Service [Servicio Galego de Saúde/SERGAS], A Coruna, Spain; 9 Catalonian Health Institute, Institut Català de la Salut/ICS, Barcelona, Spain; 10 Department of Health, Catalonian Public Health Agency, Catalonian Regional Authority [Generalitat de Cataluña], A Coruna, Spain; 11 Ribeira Health Center, Galician Health Service, Ribeira, Spain; 12 Santiago de Compostela University Clinical Teaching Hospital, Galician Health Service, A Coruna, Spain

**Keywords:** Pregnant individuals, Smoking cessation, Alcohol consumption, Cannabis, Qualitative research

## Abstract

**Background:**

Despite multiple recommendations and strategies implemented at a national and international level, cigarette smoking, alcohol consumption, and cannabis use during pregnancy remains high in most countries. The objective of this study was to examine key stakeholders’ perception of the treatment interventions adopted in Spain, to identify political, organizational and personal factors associated with successful implementation, and to propose strategies for improvement.

**Methods:**

A qualitative study with a phenomenological approach was conducted in 2022. The target groups were: (1) clinical decision makers in the field of addiction science, (2) health professionals who carry out treatment interventions, and (3) pregnant individuals who use tobacco, alcohol or cannabis. Two focus groups and eight in-depth interviews were conducted, recorded, and transcribed. Exploratory analysis and inductive open coding was performed, codes were merged into categories, and subcategories were identified.

**Results:**

The analysis resulted in 10 subcategories which were further merged into three main categories: (1) Degree of adoption and utility of treatment interventions implemented; (2) Needs and demands with respect to the organization of treatment interventions; and, (3) Personal barriers to and facilitators for treatment. Respondents reported that despite multiple national and regional cessation initiatives, treatment interventions were rarely adopted in clinical practice. Health care administrators demanded reliable records to quantify substance use for better planning of activities. Health care professionals advocated for additional time and training and both echoed the importance of integrating cessation interventions into routine prenatal care and creating in-house specialized units. The difficulty in quitting, lack of awareness of risk for foetus and child and the controversial advice were identified as barriers by pregnant individuals.

**Conclusions:**

Consistent with previous work, this study found that cessation strategies implemented by the health authorities are not effective if they are not accompanied by organizational and behavioral changes. The current study identifies a set of factors that could be pivotal in ensuring the success of treatment interventions targeting tobacco, alcohol and cannabis use among pregnant individuals.

**Supplementary Information:**

The online version contains supplementary material available at 10.1186/s12889-024-18397-x.

## Background

Tobacco smoking, alcohol consumption and cannabis use during pregnancy remains an important public health problem that affects maternal-fetal health [1]. Preterm birth, miscarriage, sudden infant death syndrome, and alterations in children’s cognitive development are some of the negative outcomes associated with the use of these substances [2–6].

The 2014 Surgeon General’s report [3] found the scientific evidence sufficient to infer a causal relationship between smoking during pregnancy and low birth weight, fetal growth restriction, premature rupture of membranes, placenta previa, placental abruption, premature delivery, reduced pulmonary function in infants, sudden infant death syndrome and orofacial clefts in offspring. Alcohol consumption has also been associated with several of these adverse pregnancy outcomes, including stillbirth, spontaneous abortion, premature birth, and intrauterine growth retardation. Moreover, alcohol consumption during pregnancy is a recognized cause of fetal alcohol syndrome (FAS) [7, 8], having children born to mothers who drink a higher risk of experiencing behavioral, cognitive, and learning difficulties, as well as speech and language delays, even in the absence of the typical facial and physical features associated with FAS [9]. Concerning cannabis, it has been mostly linked to the risk of preterm delivery, having a small-for-gestational-age baby, or being admitted to the neonatal intensive care unit [10], although some studies conducted in humans and animals suggest that it may also lead to alterations in offspring [11–14].Although the prevalence of cigarette smoking in pregnant individuals has decreased in middle and high income countries, this decline appears to have slowed down in the second decade of the 21st century. According to a 2018 meta-analysis [15], the prevalence of self-reported tobacco smoking in European pregnant individuals is estimated at 8.1% and the prevalence of alcohol use, at 9.8%. In countries such as Spain and Ireland, the prevalence of smoking in pregnant individuals exceeds 20% [16] and while estimates of alcohol use vary widely across studies, some show an alarmingly high prevalence of alcohol use during pregnancy confirmed by biomarkers (62,7%) [17]. The estimated worldwide prevalence of cannabis use during pregnancy stands at 5% [18], and is strongly associated with tobacco use [19]. It cannot be overlooked that polysubstance use during pregnancy is usual, notably with alcohol, marijuana and tobacco (20,21). Polysubstance use during pregnancy increases the risk or severity of adverse maternal and infant outcomes compared with single-substance use.

It is important to acknowledge that Spain, like many other mediterranean countries, has a long-standing drinking tradition. To reduce alcohol-related harm, especially among young people, the public consumption of alcohol has been prohibited in most Autonomous Communities, except in designated areas like terraces [22]. Advertising of high-proof alcoholic beverages with an alcohol content of over 20% has also been banned on television, internet, digital media and in some Autonomous Communities, this prohibition extends to the public highway [23]. The labeling regulation for alcoholic beverages was also recently amended by Regulation (EU) 2021/2117. The regulation mandates that all products with more than 1% alcohol must present nutritional information, a list of ingredients, and information on allergen content on their label. Nevertheless, unlike tobacco labeling, there is no inclusion of health effects information for any demographic group. It is noteworthy that some programmes associated with the alcohol industry like the “Wine in Moderation Programmes”, caution against the consumption of wine and other alcohol beverages during pregnancy.

The legislation pertaining to tobacco consumption are much stronger. Since 2011 Spain has in place a national law (Law 42/2010, Official State Bulletin, 2010) that addresses the health measures against smoking and the legislating concerning the sale, supply, consumption and advertising of tobacco products. Currently, smoking is prohibited in all closed spaces and the law extends the ban to outdoor spaces such as schools, health centers, and areas of children’s parks and playgrounds. All forms of tobacco advertising are prohibited across all media channels, including programs featuring hosts, collaborators or guests engaging in smoking or mentioning, directly or indirectly brands, tradenames, logos or any other identifiers linked with tobacco products. In addition, among the 14 text and pictographic warnings displayed on tobacco packaging [24], there is a specific message targeted at pregnant individuals: “Smoking can kill the unborn child.” According to the results of a survey of tobacco control activities carried out in 37 European countries with the Tobacco Control Scale in 2021 [25], Spain ranked 11th among the 37 European countries in terms of Tobacco Control legislation.

Concerning cannabis, the consumption is current prohibited by law in many countries, including Spain, and this could probably explain the lower rates in relation to the other two substances. However, the prevalence of cannabis use could be liable to change if the cannabis is legalized, especially given the significant increase in usage in countries like the United States of America (USA) [26] and European countries, such as France [27, 28] following recreational legalization. Studies conducted in the USA [29, 30] have shown that in states where cannabis has been legalized, there is an increased social acceptance of cannabis use and a diminished risk perception [31]. There is a growing concern that this growing acceptability could extend globally, highlighting the need for further research on this topic.

Despite the legislative measures and the numerous guidelines offering recommendations regarding interventions for substance cessation at both a national and international level [32], the use of these substances during pregnancy remains an important challenge. Several studies [33, 34] have highlighted a diminished perception of risk as a factor associated with quitting substance use. In relation to tobacco, some studies suggest that many pregnant individuals still believe that the stress of quitting smoking outweighs the risk of tobacco use [35, 36]. Regarding cannabis, it has been reported that some women view it as beneficial for alleviating nausea during the first trimester of pregnancy [37, 38]. Moreover, research on pregnant individuals’s perception of risk regarding the consumption of low-alcohol beverages like wine and beer during pregnancy has suggested that such concerns are virtually absent among pregnant individuals [39]. Nevertheless, overall research is scarce and some of the existing studies may be outdated, considering the legislative and societal changes over the past few decades. Exploring the prevailing opinions of the key stakeholders concerning these issues could provide important insights into the current factors contributing to the lack of effectiveness of cessation strategies. The main aim of this study was firstly, to examine the perceptions and experiences of health professionals, managers and administrators, as well as pregnant individuals and their partners, regarding the treatment interventions and identify the barriers to, and facilitators of, implementation of cessation strategies.

The findings from this study may help identify areas for improvement and enhance prenatal care guidelines.

## Methods

### Design

We conducted a qualitative study using a phenomenological approach. Data collection took place in Santiago de Compostela (Spain). Results have been reported in accordance with Standards for Reporting Qualitative Research [40].

The target groups were: (1) healthcare managers and administrators responsible for implementing health policies in the field of addictive substance treatment; (2) health professionals tasked with carrying out treatment interventions; and (3) pregnant individuals who smoked tobacco, consumed alcohol or used cannabis.

Health professionals and managers/administrators were intentionally sampled, targeting those who provided healthcare services to pregnant individuals (gynecologists and obstetricians, midwives, nurses, pharmacists, psychologists and social workers). Decision makers at both macro-and micro-level (specialists in mental health and addiction science management, managers/administrators of drug rehabilitation and planning centers) were also included. Pregnant individuals who were users of tobacco, alcohol or cannabis were identified by two primary care midwives during routine prenatal care visits conducted as part of the follow-up schedule for pregnant individuals in primary care. Pregnant individuals were inquired about their use of tobacco, alcohol and cannabis thorough a broad question “since you are pregnant, have you drunk any type of alcohol, including wine or beer, or smoked cigarettes or cannabis?”. Those who openly admitted to consuming either tobacco, alcohol or cannabis and agreed to participate, had their contact details shared with the research team, and subsequent interviews were carried out until saturation was reached.

### Field work

Data collection took place from June through September 2022. We formed two focus groups, one comprised managers/administrators (n = 8) and the other consisted of health professionals (n = 9). Specific moderator’s guide weredeveloped for each group. To gather information from pregnant individuals (n = 6) who either used tobacco, alcohol and/or cannabis, we opted for in-depth one-on-one semi-structured interviews. We chose this technique with the aim of minimizing social desirability biases and fostering participation, as smoking, alcohol consumption and/or cannabis use during pregnancy are sensitive topic that can lead to concealment. Pregnant individuals were compensated with 25€ for their participation in the interview.

The moderator’s guide used in both the focus groups and in-depth interviews (Supplementary File 1) was collaboratively developed by members of the research team including a psychologist, an epidemiologist and a midwife (YTP,LVL and AVF), along with two moderators (both experts in qualitative methodology, one from the research team, YTP).Subsequently, all members of the research team reviewed them in order to detect errors and clarify the more complicated questions. These were piloted in 3 smoking non-pregnant individuals before data collection, no modifications were made to the interview guides.

The focus groups were conducted by expert professionals, with two research team observers present (AVF and LVL). Data collection continued until saturation in the discourse was achieved. The group sessions and interviews were recorded and transcribed after obtaining the participants’ prior written consent.

### Data-analysis and triangulation

We used a double triangulation approach to address completeness, convergence, and dissonance of key themes. Data-source triangulation was achieved by contrasting the information coming from different stakeholders (managers/administrators, health professionals, and smoking pregnant individuals and their smoking partners when volunteering to participate), and by using purpose-designed tasks to complement the participants’ discourse. For the analysis, the following steps were followed: (1) transcription of the data, and reading and rereading to become familiar with the material; (2) exploratory analysis of data and open inductive coding; (3) merging of similar codes into categories; (4) analysis of the data, coding into categories and subcategories; and (5) drafting of the results report. Subsequently, all research group members participated in a review of the categories and subcategories identified, and provided their insight. Differences of opinion were discussed until consensus was reached.

### Ethical aspects

The study was approved by the Research Ethics Committee of Lugo-Santiago (registration code 2021/402). All participants received a briefing note outlining the purpose and methodology of the study, and gave their prior written informed consent.

## Results

A total of 23 individuals (9 professionals, 8 managers/administrators, 6 pregnant individuals) participated in the study. The professional focus group session lasted for 1 h and 30 min, while the session with managers/administrators lasted 1 h and 15 min. The in-depth interviews with the participants ranged from 20 to 25 min each.

Table 1 shows the main characteristics of the participants.

**Table 1:** Characteristics of participants

	CLINICAL HEALTH PROFESSIONALS Focus Group	MANAGERS/ ADMINISTRATORS Focus Group	PREGNANT INDIVIDUALS Semi-structured 1:1
N [TOTAL *N* = 25]	9	8	6
WOMEN/MEN	8/1	4/4	6
**MEDIAN AGE IN YEARS**	**42** (37-50)	**56** (45-62.25)	**34.5** (32.5-37.25)
**EDUCATIONAL LEVEL**	**University**	**University**	**-pregnant individuals**: 2 university, 2 secondary, 2 primary
**PROFESSIONAL PROFILE OF ADDICTION SCIENCE, PREGNANCY AND/OR HEALTHCARE MANAGEMENT** **SPECIALTY FIELDS**	**-Gynecologist-obstetrician**: 2 **-Psychologist**: 1 **-Pharmacist**:1 **-Midwife**: 3 (1 representative Spanish Federation of Midwives; 1 representative Galician Association of Midwives). **-Social worker**: 1 **-Community Health nurse**: 1	**-Drug rehabilitation unit**: 2 **-Healthcare planning**: 1 **-Prevention of addictive behaviors**: 2 **-Mental health**: 1 **-Prevention and control of smoking**: 1 **-Epidemiologist**: 1	**N/A**
**MEDIAN YEARS OF WORK EXPERIENCE** [INTERQUARTILE RANGE Q1-Q3]	**15** [10–28]	**20.5** [20–30]	**N/A**

### Thematic analysis

In total, 10 subcategories were identified and merged into the following 3 main categories: (1) degree of adoption and utility of treatment interventions implemented; (2) needs and demands of decision makers and clinicians with respect to the organization and delivery of the treatment interventions; and, (3) personal barriers to and facilitators for treatment.

### Degree of adoption and utility of treatment interventions implemented

#### Implementation of cessation interventions

In the managers/administrator group a theme emerged surrounding the multiple regional and national activities put in place to address substance cessation during pregnancy, particularly focusing on tobacco (Cessation guidelines and treatment protocols, electronic-record information integration, etc.). However, several of the participants acknowledged doubts regarding the implementation of these guidelines and protocols in clinical practice. Sentiment was corroborated by all health professionals, who consistently recognized that the adoption was very much professional dependent. All pregnant individuals agreed on the lack of support for substance cessation, at most they were just offered a very generic list of recommendations.*Manager/administrator 6: “In the National Health System we’ve got a whole lot of plans, protocols and so on. But then in practice they’re not implemented. “*.

*Health professional 6: “What we find in our area is a fairly marked lack of interest at a governmental level to implement use-cessation policies.”*

*Pregnant woman 2: “At the gynecologist´s examination they do tell you, but they give it to you like a list of very generic recommendations, and naturally without insisting on any particular aspect.”*

### Needs and demands of decision makers and clinicians with respect to the organization and delivery of the treatment interventions

Data on substance use: Stakeholders belonging to the managers/administrators group agreed that there was a great lack of knowledge on the part of the healthcare authorities about the real use of these substances in pregnancy, and that this constituted a major obstacle to the planning of activities in this sphere. The normalization of consumption, concealment secondary to fear of prejudice, and poor health records were the main reasons articulated.

*Manager/administrator 1: “How many women consume alcohol during pregnancy? We have no idea. Because, it’s something that’s very normalized but it’s not really approved of when you’re pregnant.”*

#### Time allotted to medical visits

All health professionals and some managers echoed concerns regarding the lack of time for carrying out cessation interventions. Reasons cited included the high volume of pregnant individuals assigned per working day and the need to address counseling on other risk factors. Several of the interviewed health professionals said that they would require additional time to perform cessation interventions. *Health professional 1: “If they gave me two mornings a week to devote myself to women smokers, to obesity and any other problem, that would be the ideal thing, but we do what we can.”*

*Manager/administrator 8: “The role of midwives in health promotion is indisputable. Quite another thing is their capacity in terms of schedules, given the current situation of the National Health System -often on the verge of collapse.”*

#### Need for training

The common belief among the managers/administrators was that all healthcare professionals attending to pregnant individuals possessed the training and skills for advising on drug cessation. However, health care professionals consistently countered this, stating that they lacked training, skills, know-how or time to conductindividual cessation interventions effectively. They also expressed doubts about the impact of solely providing information and advice.

*Manager/administrator 1: “All the professionals that attend pregnant women are trained to give the same recommendation, which is to quit their use of psychoactive drugs”*.*Health professional 2: “The problem is that we aren’t trained. So, the time comes, and most likely it’s not so much that you don’t want to intervene. Most probably it’s just that you don’t know how to intervene.”*

Creation of specific treatment units for pregnant individuals: A broad consensus emerged regarding the importance of integrating cessation interventions into routine prenatal care. Integrating cessation interventions into routine care would avoid increasing the number of medical visits and save time and money. Most health professionals called for specialized staff and the creation of specific cessation of in-house units for pregnant individuals. They argued that they lacked the know-how or time and that there were currently no suitable facilities to send pregnant individuals who smoke tobacco, or are occasional consumers of alcohol or cannabis. On the contrary, administrator and managers strongly emphasized that all professionals who attend pregnant individuals should be capable of providing parallel counseling, although they all recognized the need for dedicated in-house units at the respective health centers to approach complicated cases.

*Health professional 4: “We need to have dedicated units at the centers that can intervene. It’s no use recommending people to quit smoking, and it’s not much use handing out a brochure or pamphlet. What is of use is time, and making an individualized plan for each patient.”*

*Manager/administrator 3: “So I think that midwives and any other health professionals who see them should obviously take advantage of this window of opportunity to ask if they’re using any of these substances, and if that’s the case, to give them simple, clear, personalized advice that they can understand.*

### Personal barriers to and facilitators for treatment

Difficulty of quitting tobacco: Several of the pregnant individuals mentioned that they found it very difficult to quit smoking. Stress was identified as one of the main barriers to quit smoking by pregnant individuals.

*Pregnant woman 1: One shouldn´t smoke but its very difficult to control. I wanted to quit smoking before becoming pregnant and I cut down a lot. I always thought that when I got pregnant it was going to be easy, because then you don´t stop smoking for yourself but for the one that´s coming, but… sometimes it makes you so nervous that it´s really difficult to quit”.*

Concealment of behaviors: Most health professionals perceived that pregnant woman commonly conceal the use of these substances, which was also mentioned by many pregnant individuals. All said that they felt judged for smoking, consuming alcohol and/or using cannabis during pregnancy.

*Health professional 6: *“*They probably hide the fact that they´re smoking from their family circle and their friends. In general, at other times of life there isn´t the same perception of harm but at this time, they are exposed to the judgement of others. I think they often conceal it from health professionals as well*”.

*Pregnant woman 2: “If that person –referring to a gynecologist- asks me if I smoke, I’d say no”.*

Low perception of risk: Both health professionals and managers/administrators felt that pregnant individuals had a low perception of the risks associated with these substances, which they identified as a major barrier to addressing this issue. The low perception of the risks associated with these substances was corroborated by most pregnant individuals. In the case of tobacco, they conveyed a mixed understanding and awareness of the consequences of smoking.While acknowledging its harmful nature, they expressed uncertainty regarding the specific negative effects of tobacco use, and did not remember being provided with detailed information by their healthcare provider. With regards to alcohol, pregnant individuals expressed a lack of perception of risk associated with sporadic consumption, particularlyregarding consumption of low alcohol content beverages such as wine and beer. A number of them mentioned “fetal alcohol syndrome”, though they were unaware of its consequences and believed it only occurred with high alcohol consumption. 

*Health professional 5: “They don´t stop seeing women around them who admit that they used to smoke and that absolutely nothing happened during pregnancy…”*.

*Pregnant woman 4: “I never drank alcohol during pregnancy but they say that having a drink now and then isn´t bad. Not drinking every day or going for beers every day, but one beer or so can do no harm.”*

Smoking reduction counseling: Gynecologists/obstetricians and midwives found it unlikely that health care professionals would advise patients to merely cut down rather on substance use rather than quit completely. Conversely, managers/administrators and health professionals from other specialties believed that pregnant individuals were still recommended to reduce rather than to quit altogether to avoid the effects of the mother’s stress on the unborn child. Most pregnant individuals and their partners shared this latter opinion.

*Health professional 2: “In all the departments in which I have worked, you´re not going to hear about a single gynecologists telling a patient that the anxiety caused by quitting smoking is worse than having a cigarette”.*

*Pregnant woman 5: “I heard it myself, nobody told me but I heard that sometimes it´s better to smoke than go through the withdrawal syndrome”.*

Pregnant individuals’s motivation: Insofar as smoking was concerned, professionals perceived pregnant smokers and partners as having a special motivation to quit, particularly strong in primiparous individuals. Some pregnant participants explicitly mentioned this enhanced motivation to quit all substance use during pregnancy.

*Health professional 8: "In women who haven´t had children previously the motivation is stronger than in those who have had children previously and smoked and nothing happened”.*

*Pregnant woman 6: “Apart from the fact that I didn’t feel like it, I certainly thought a lot about the baby. I knew that if I did smoke, it could harm him, it was a thought that just came to me”.*

## Discussion

The main aim of the study was to conduct a critical analysis of the factors that can influence tobacco smoking, alcohol consumption and cannabis use during pregnancy in Spain, by including the perspective of health professionals, managers/administrators, and pregnant individuals. To our knowledge, this is the first study to explore this from three perspectives: management, clinical practice and pregnant individuals.

The results show that despite the various strategies implemented by health authorities, the level of implementation remains low. Consistent with findings from other studies [40–43], we identified the lack training, time and skills as barriers to the adoption of treatment interventions [44, 45]. Our study, like another one performed in the United Kingdom, revealed that midwives often lacked confidence intheir ability to undertake treatment interventions [42], possibly due to the absence of institutionalized training in this regard. Lack of confidence of midwives to carry out treatment interventions underscores the need to advocate for improved training for all personnel responsible for the care of pregnant individuals regarding the risks of substance consumption during pregnancy. One potential approach could involve training addiction referral staff in maternity wards. It should be noted that the functions performed by midwives in the different countries across Europe are similar, due to the fact that it is an officially regulated profession [46]. Our findings suggest that the best approach would be for the health professionals themselves to be capable of providing access to effective cessation interventions, thereby minimizing the need for additional visits. However, participants expressed the belief that dedicated units might be needed to treat difficult cases. Midwives mentioned that currently faced uncertainty regarding where to refer pregnant individuals who smoked tobacco, or were occasional consumers of alcohol or cannabis, as they did not view the existing addictive behavior units as suitable for pregnant individuals. A study in Ohio, where 14% of midwives reporting not knowing where to refer pregnant smokers experiencing withdrawal symptoms and requiring nicotine patch use [43].

This study identified various personal barriers to and facilitators for cessation from the perspective of the pregnant individuals. One of the barriers identified was concealment due to fear of being judged. Current evidence suggests that feeling judged can lead to feelings of guilt, resulting in depressive symptoms, and difficulties in quitting smoking [47].. The number of individuals concealing substance use is estimated to be high. A study conducted in the USA, which included 4,197 pregnant individuals aged 20 to 44 years, found that 23% of pregnant individuals who were smokers did not acknowledge smoking [48]. Another study, also conducted in the USA, which analyzed the urine of 53 children between the ages of 0 to 3 years, found that 20.8% had marijuana metabolites detectable in urine [49]. On the contrary to what might be expected, our study also found that pregnant individuals were only vaguely aware of the risks of alcohol consumption, aside from fetal alcoholic syndrome [50], which they attributed to chronic and heavy alcohol use. The perception of low risk is in alignment with other studies, such as the study conducted in 2013 and 2014 in Sweden and England involving 43 pregnant individuals and their partners [51]. In this study the participants doubted that “low-to-moderate” alcohol consumption might pose health risks. A systematic analysis of 694 data sources and 592 prospective and retrospective studies on alcohol consumption in the general population, also indicated that overall, the perception was that low alcohol consumption posed no harm to one´s health at any stage of life [52].

Our results suggest that the perception of risk and motivation to quit is higher in primiparous individuals than in multiparous individuals who have smoked in previous pregnancies. This finding is in line with the results of a systematic review of 54 studies involving 505,584 individuals, which found that pregnant primiparous individuals had a higher likelihood of quitting smoking than did multiparous individuals (OR: 1.85; 95%CI: 1.68–2.05) [53].

We also identified that individuals still hold the belief that the stress of quitting is worse than continuing smoking during pregnancy. This notion has also been observed in previous qualitative studies [35, 36]. However, existing evidence for general population shows that mental health does not worsen as a result of quitting, but rather improves a small-to-moderate amount [54].. These findings are important because they highlight the important misinformation that exists on this issue, which has been echoed in the Spanish social networks [55].

Barriers reported in our study, such as low perception of risk, advice to cut down smoking or the difficulty of quitting, have also been observed in many other countries [35, 39]. Different materials have been elaborated to support health professionals to convey counseling on tobacco smoking among pregnant individuals [56]. However, with respect to alcohol, a systematic review published in 2022 concluded that the information on alcohol consumption in pregnancy should be improved qualitatively and quantitatively [57].

The main facilitator identified for quitting all the three substances was the motivation to safeguard the health of their offspring. Motivation to safeguard the health of their offspring has also been found in other studies [58], being commonly acknowledged that the perinatal period is a time when pregnant individuals are highly motivated to change unhealthy behaviors (59). A previous qualitative study conducted in the United Kingdom, which recruited 12 pregnant smokers, recorded the testimony of one participant who cited pregnancy as the sole reason for quitting smoking (60). This study has several limitations; the first being the inclusion of pregnant individuals from only one Spanish Region. Additionally, there is a gap between the discourse of professionals on the three substances and that of pregnant individuals, since none of the participants acknowledged using cannabis and only one admitted to using alcohol. However, based on the fact that interviewed pregnant individuals themselves commonly acknowledge that pregnant individuals conceal alcohol and cannabis use due to fear of judgement and legal issues, we believe that the participants may be representative of what would be expected in real practice. It is worth mentioning that the discourse of pregnant women completely coincided, which is why only six pregnant individuals were interviewed.

Another potential limitation of our study is the reliance on self-reported information about psychoactive substance use during pregnancy; however, previous studies have demonstrated the reliability and accuracy of such self-reported information, with validity extending up to 11 years postpartum (61, 62). We acknowledge that the accuracy of this information may be influenced by several factors, including the nature and setting of the interviews, which are conducted retrospectively after childbirth (63). The possible influence of these factors underscores the need to carefully consider the limitations associated with the authenticity of data obtained through interviews, recognizing the potential impact of various factors on the accuracy of participants’ recall of their behaviors. The study also has strengths: to our knowledge it is the first study to jointly address the perspective of managers/administrators, health professionals, pregnant individuals with respect to smoking, alcohol consumption, and cannabis use during pregnancy.

## Conclusions

In summary, the results suggest that the extent of smoking, alcohol consumption, and cannabis use during pregnancy might be underestimated due to individuals’s low perception of risk and concealment of their own consumption and/or use, particularly, in the case of cannabis. Similar to previous studies, this study highlights the fact that strategies implemented by the health authorities might not be effective if they are not accompanied by structural and behavioral changes. It also emphasizes the misinformation that exists regarding the risks associated with the use of these substances.

### Electronic supplementary material

Below is the link to the electronic supplementary material.

Supplementary Material 1

## Data Availability

All data generated or analysed during this study are included in this published article (and its supplementary information files).
