# Supplementary material for: Perception of pregnant individuals, health providers and decision makers on interventions to cease substance consumption during pregnancy: a qualitative study
Source: BMC Public Health. 2024 Apr 9;24:990. doi: 10.1186/s12889-024-18397-x (PMC11003004; doi:10.1186/s12889-024-18397-x)
Supplement: Supplementary file 1 — Supplementary Material 1 [file 12889_2024_18397_MOESM1_ESM.docx]

**SUPPLEMENTARY FILE 1**

**TOPIC GUIDE: FOCUS GROUP OF HEALTH PROFESSIONALS**

- One of the avoidable risk factors in pregnancy is drug use, how prevalent do you perceive this reality to be?; what substances are most used by women during pregnancy?; do you think that use is increasing or decreasing in comparison with previous years?: which variables do you think are associated with drug use in pregnancy?
- Do pregnant women have the necessary information about the harmful effects that drug use during pregnancy can have for development of the fetus?; what reasons account for the fact that, despite this, such use often continues to occur?
- What does routine public healthcare in Spain mean in terms of promoting cessation of drug use (specifically, tobacco, alcohol and cannabis) in pregnant women?; are there differences by Autonomous Region/health area or even among different professionals?; is there a common protocol?
- What is the current role of primary care midwives when it comes to promoting cessation of drug use in pregnant women (in general, and specifically tobacco, alcohol and cannabis)?; what do you think it should be?
- Do you know of other interventions undertaken in other countries for promotion of cessation of drug use in pregnant women?; what do they consist of?; do you think it would be of interest to implement something like that in public healthcare in Spain?
- Would applying a cessation intervention in public healthcare in Spain be feasible?; what resources would be needed to implement it?; would it be viable to obtain them?

**TOPIC GUIDE FOCUS GROUP OF MANAGERS/ADMINISTRATORS**

• One of the avoidable risk factors in pregnancy is smoking, alcohol consumption and cannabis use. Based on the data you know and handle, how prevalent is this reality?; do you consider it a problem that requires a priority approach?; how high a priority do you think it is?

• Do you have information on what routine public healthcare in Spain means in terms of promoting drug use cessation (specifically tobacco, alcohol and cannabis) in pregnant women?; are there differences by Autonomous Region/health area or even between different centers?; is there a common protocol?

• Do you know of other interventions undertaken in other countries for the promotion of drug use cessation in pregnant women?; what do they consist of?; do you think it would be of interest to implement something like that in public healthcare in Spain?

• Do you know about any new approach for this purpose?, some intervention with a novel aspect that could be implemented? (this can be skipped, depending on the time of the other replies)

• How do you assess the impact of smoking, alcohol consumption and cannabis use during pregnancy?, and specifically the impact on the health system?; how do you assess the use of resources that this entails?

• What is the current role of primary care midwives when it comes promoting cessation of smoking, alcohol consumption and cannabis use in pregnant women?; what do you think it should be?

**IN-DEPTH INTERVIEWS OF PREGNANT WOMEN**

- What do you think about drug use in pregnancy? And tobacco/alcohol/cannabis use in particular?
- Do you think it is a real problem right now?
- At present, do you use any drug, and specifically tobacco/alcohol/cannabis?
- Have you quit smoking/alcohol/cannabis because of the pregnancy? What was your reason for quitting?
- Does your partner smoke/consume alcohol/use cannabis?
- Has your partner quit tobacco/alcohol/cannabis because of wanting to become or becoming pregnant? Do you think that the fact that her partner quits helps the pregnant woman to quit as well?
- If you use or know someone who uses these drugs, what are the reasons why you continue to smoke/consume alcohol/use cannabis in pregnancy?
- Do you think that women feel judged for smoking/consuming alcohol/using cannabis during pregnancy? Do you have any personal experience of this?
- (if the partner smokes): Do you think that people will judge him/her for smoking while you are pregnant?
- During the pregnancy, has anyone (whether or not a health professional) advised you to quit tobacco/alcohol/cannabis? Who?
- Of these persons, has anyone offered to help you quit smoking? Who? What type of help? What is your perception of the help you received?
- Have you ever tried to quit or thought about quitting? Why?
- Do you think that you would be able to quit whenever you wanted?
- Now I’d like you to talk to me about the time before you were pregnant. I imagine that, like everyone else here in Spain, you used to have a beer, glass of wine, a drink or two (if you say you did). Do you miss it?
- For instance, if you go out on Saturdays with your women friends/partner, do you find it hard not to drink, not even a quick beer?
- It is clear that in Spain alcohol is used a lot to socialize, and I understand that it is difficult. Do you think that having a beer or glass of wine during your pregnancy poses any risks for your baby?, and what about a rum and coke? What risks?
- Good, coming back to smoking, do you think that it would be better for you to quit tobacco/alcohol/cannabis little by little or all at once?
- Do you think that it would be better for the baby if you quit tobacco/alcohol/cannabis little by little or all at once?
